# Impact of lymphocyte transformation test on the diagnostic accuracy of the culprit drug in drug-induced cytopenias: a case-control study

**DOI:** 10.3389/fphar.2025.1601450

**Published:** 2025-12-05

**Authors:** Susana Martín-López, Olga Rogozina, Daniela Aguilar-Concepción, Miguel Álvarez-Montero, Ramón Pardo-Puras, Ibtissam Akatbach-Bousaid, María Jiménez-González, Ana Martínez Feito, Miguel González-Muñoz, Elena Ramírez

**Affiliations:** 1 Clinical Pharmacology Department, La Paz University Hospital-IdiPAZ, Faculty of Medicine, Autonomous University of Madrid, Madrid, Spain; 2 Immunology Department, La Paz University Hospital-IdiPAZ, Madrid, Spain; 3 Clinical Trial Unit, La Paz University Hospital-IdiPAZ, Madrid, Spain

**Keywords:** drug-induced cytopenias, adverse drug reactions (ADR), idiosyncratic drug-induced neutropenia (IDIN), idiosyncratic drug-induced agranulocytosis (DIAG), drug-induced immune haemolytic anaemia (DIIHA), causality assessment algorithms, delayed hypersensitivity reactions, lymphocyte transformation test (LTT)

## Abstract

**Background:**

Drug-induced cytopenias are serious adverse drug reactions (ADRs), often challenging to diagnose. While causality algorithms offer high sensitivity and positive predictive values, they exhibit low specificity and negative predictive values for identifying the causative drug(s). Therefore, complementary diagnostic tools are required.

**Objective:**

This study aimed to evaluate the utility of the lymphocyte transformation test (LTT) in supporting the causality assessment of the Spanish Pharmacovigilance System (SPS) causality algorithm in the diagnosis of the implicated drug(s) in drug-induced cytopenias, using a sample of 40 cases and 85 controls.

**Methods:**

Suspected cytopenia cases were identified through the Proactive Pharmacovigilance Program for Laboratory Signals in Hospital or via pharmacovigilance consultation. Control patients completed their treatment without experiencing any ADR. A receiver-operating characteristic (ROC) curve analysis was performed to determine the optimal stimulation index (SI) cut-off for the LTT, maximizing the sum of specificity and sensitivity values to accurate identify cytopenias cases.

**Results:**

The case group included 29 cases (72.5%) of agranulocytosis, 6 (15.0%) of neutropenia, 2 (5.0%) of haemolytic anaemia, 2 (5.0%) of bicytopenia and 1 (2.5%) of bone marrow aplasia in 39 patients. Most had ≥3 comorbidities (66.7%) and no previous allergies (71.8%). Eighty-four drugs were suspected as causative agents (SPS-score ≥+4), with metamizole being the most frequent (17.2%), followed by acetaminophen (9.1%) and amoxicillin-clavulanate (8.1%). Eight cases (20.0%) involved a single suspected drug, while two cases (5.0%) involved polypharmacy (≥5 drugs). LTT was positive in 75% of cases and in 1.2% of controls. Forty one (41.4%) of the 99 suspected drugs yielded positive LTT result. With an optimal SI cut-off of 1.95, the LTT achieved a sensitivity of 72% and a specificity of 99% (area under the curve, 0.86; 95% CI 0.77–0.96; *p* < 0.001). With monitoring, drug re-exposure was fully tolerated in patients with negative LTT results (100%), but poorly tolerated in one-third of those with positive LTT results. A causality score below 6 and a negative LTT yielded a 100% negative predictive value for drug tolerance (95% CI: 94.4%–100%).

**Conclusion:**

This study demonstrates that the LTT can be a valuable tool for strengthening causality assessment in suspected drug-induced cytopenias.

## Introduction

The term *blood dyscrasias* encompasses a diverse group of disorders affecting blood components, including red blood cells (RBCs), white blood cells (WBCs), neutrophils, platelets, the plasma constituents, lymph tissue, bone marrow, and blood vessels (Thachil and Bates, 2017). These disorders can manifest as either an increase or a decrease in specific type cell types, nor multiple cells lines. Blood dyscrasias characterized by a deficiency of blood cells include anaemia (decrease of RBCs), leukopenia (deficiency of WBCs) and thrombocytopenia (decrease of absolute platelet count, APC). Pancytopenia refers to a condition characterized by a deficiency in all major blood cell types.

Potential causes and risk factors for blood dyscrasias are varied, spanning infections, environmental exposures, autoimmune or malign conditions, genetic predispositions, vitamin and mineral deficiencies and medication-induced effects (drug-induced cytopenias).

Leukopenia, defined as a WBC count under 4000 cells/mm^3^, encompasses various deficiencies based on WBC type (Tigner et al., 2022). Neutropenia, a reduction in the absolute neutrophil count (segmented cells and bands), the most abundant WBC, is specifically defined as an absolute neutrophil count (ANC) under 1500 cells/mm^3^ after the first year of life (Boxer, 2012; Connelly and Walkovich, 2021; Neutropenia, 2024). Drug-induced neutropenia is caused by decreased production or increased destruction of neutrophils, often due to chemotherapeutic agents suppressing bone marrow myeloid progenitor cells. Non-chemotherapy idiosyncratic drug-induced neutropenia (IDIN) is a rare but potentially lethal condition, with an estimated incidence of 2.4–15.4 cases per million people (Curtis, 2014; Johnston and Uetrecht, 2015; Andrès et al., 2017; Curtis, 2017). Specifically, *agranulocytosis* (or *severe neutropenia*) is defined as a profound decrease in circulating neutrophil count, below 500 cells/mm^3^ (Medrano-Casique et al., 2016; Curtis, 2017). Individuals with an ANC under 100 cells/mm^3^ face a high risk of infection-related morbidity and mortality (Curtis, 2017). Epidemiological studies show variations across racial groups (Zhou et al., 2023), with an estimated prevalence of approximately 35.5 million people in the United States (Zhou et al., 2023) and an annual incidence approximately 1.6–15.4 cases per million population (Curtis, 2014; Andrès et al., 2017). Causes of neutropenia/agranulocytosis include viral, bacterial or parasitic infections, blood disorders, autoimmune neutropenia, toxic agents, splenomegaly, cirrhosis, mitochondrial disease, cyclic neutropenia, benign chronic neutropenia, and age below 3 months (Curtis, 2017). While IDIN is not the most common cause, it should be considered in unexplained neutropenia (Curtis, 2017). Drugs account for two-thirds to three-quarters of severe neutropenia/agranulocytosis cases (Ibáñez et al., 2005; Medrano-Casique et al., 2015; Johnston and Uetrecht, 2015; Curtis, 2017). Common drugs associated with neutropenia or agranulocytosis include clozapine, dipyrone, diclofenac, spironolactone, antithyroid drugs, carbamazepine, sulfamethoxazole-trimethoprim and vancomycin (Curtis, 2014; Medrano-Casique et al., 2016).

IDIN develops when drug-dependent antibodies target neutrophil membrane glycoproteins, leading to destruction of neutrophils (Curtis, 2014). However, alternative mechanisms have also been proposed, including type IV delayed hypersensitivity reactions in which drug-specific T lymphocytes target mature leukocytes or their bone marrow progenitors (Posadas and Pichler, 2007). Symptoms include fever, chills, and infections, which can be fatal if untreated (Curtis, 2014). Idiosyncratic drug-induced agranulocytosis (DIAG) remains a serious adverse drug reaction (ADR) due to the high incidence of severe sepsis, including deep tissue infections, septicemia, and septic shock, in approximately two-thirds of hospitalized patients (Andrès et al., 2017). Treatment with intravenous broad-spectrum antibiotics and hematopoietic growth factors, such as granulocyte colony-stimulating factor (G-CSF), improve outcomes (Curtis, 2014; Andrès et al., 2017). With proper management, the current mortality rate is between 5% and 10% (Andrès et al., 2017).

Major types of anaemia include iron deficiency anaemia, macrocytic anaemia (caused by vitamin B12 or folate deficiency), haemolytic anaemia, aplastic anaemia and inherited conditions such as RBC enzyme deficiencies (including glucose-6-phosphate dehydrogenase deficiency and pyruvate kinase deficiency), red cell membrane diseases (such as hereditary spherocytosis and elliptocytosis) (Risinger and Kalfa, 2020) and haemoglobinopathies (e.g., sickle cell disease and thalassemia).

Haemolytic anaemia is characterized by the destruction of RBC, leading to increased lactate dehydrogenase (LDH) levels, elevated haemoglobin (Hb) catabolism with reduced Hb levels, and increased unconjugated bilirubin (UCB) levels. To compensate, bone marrow activity is increased resulting in elevated reticulocyte levels. If the underlying cause is unclear, a direct Coombs test can differentiate between immune-mediated and non-immune in origin (Baldwin et al., 2023). Drug-induced immune haemolytic anaemia (DIIHA) is an uncommon disorder primary caused by drug-triggered antibodies, which can be either drug-dependent or drug-independent (Arndt, 2014).

The development of large, computerized clinical databases linked to electronic medical records (EMRs) has significantly advance ADRs detection programs in recent decades. These programs enable clinicians to respond swiftly and appropriately. Methodologies vary considerably, tailored to the unique characteristics of each hospital. Nevertheless, they can generally be categorized into those that use sentinel words or “triggers” (e.g., “toxicity”) in EMRs and those that rely on signals from laboratory information systems (automatic laboratory signals [ALSs]). At La Paz University Hospital, pharmacological monitoring is conducted through the Proactive Pharmacovigilance Program from Laboratory Signals at Hospital, which utilizes ALSs. These signals highlight severe reactions such as agranulocytosis, aplastic anaemia, and liver injury, which are commonly associated with drug use. This program uses the causality algorithm of the Spanish Pharmacovigilance System (SPS) to diagnose the culprit drugs in cytopenias. The SPS algorithm is primarily used in Spanish-speaking environment (Aguirre and García, 2016). This program has demonstrated effectiveness in the early detection and evaluation of specific severe ADRs, achieving a sensitivity of nearly 100% and a specificity of 21.2% (Medrano-Casique et al., 2016; Ramirez et al., 2010). To improve specificity, given that re-exposure to the culprit drug is ethically prohibited, *in vitro* diagnosis techniques such as genetics, therapeutic drug monitoring, and immunological tests have been implemented.

T-cell sensitization to a drug can be assessed using the Lymphocyte Transformation Test (LTT), which measure the activation and expansion of the drug-specific memory T cells after *in vitro* incubation of the patient’s peripheral blood mononuclear cells with different concentrations of the suspected drugs (Pichler and Tilch, 2004). Current evidence suggest the LTT’s utility in diagnosing drug-induced neutropenia and agranulocytosis, particularly in patients treated with beta-lactam antibiotics, ticlopidine, pyrazinobutazone and antithyroid drugs (Sachs et al., 2000; Berg et al., 1989; Taniuchi et al., 2000; Ono et al., 1991; Kulessa et al., 1992; Maria et al., 1989; Wall et al., 1984), and only one case of aplastic anaemia diagnosed using the LTT has been reported in the literature (Saal et al., 1985; Saal et al., 1986).

Apart from haematological drug-induced cytopenias, LTT has been utilized in other drug hypersensitivity reactions and immune-mediated conditions. Various studies have demonstrated its usefulness in antibiotic allergy, including β-lactams, clindamycin, and cotrimoxazole, as well as in protein-containing drugs (Nyfeler and Pichler, 1997; Vílchez-Sánchez et al., 2020a). In addition, LTT has proven useful in the study of delayed hypersensitivity reactions induced by nonsteroidal anti-inflammatory drugs (NSAIDS), as ibuprofen or metamizole (Nin-Valencia et al., 2023). LTT has also demonstrated its usefulness in the diagnosis of delayed reactions to sulfonamides (Vílchez-Sánchez et al., 2020b) and has shown potential utility for antithyroid agents (Wall et al., 1984). LTT has proven useful to identify culprit drugs in delayed-type hypersensitivity reactions such as maculopapular exanthema, drug reaction with eosinophilia and systemic symptoms (DRESS), Stevens–Johnson syndrome, and toxic epidermal necrolysis (Pichler and Tilch, 2004; Mayorga et al., 2017; Mayorga et al., 2016; Bellón et al., 2020). Furthermore, more recent studies have shown the usefulness of LTT in drug-induced liver injury (DILI), helping to identify specific culprit drugs as antibiotics, antidepressants, antivirals, and analgesics (Rodríguez et al., 2022; Delgado et al., 2021; González-Muñoz et al., 2020), as well as excipients such as polyethylene glycols and polysorbate 80 (Rogozina et al., 2024). In this regard, LTT has also been useful in identifying immune-mediated organ-specific reactions to COVID-19 vaccines containing this type of excipients (Ruiz-Fernández et al., 2023).

The main objective of this study was to evaluate the usefulness of the LTT in supporting the causality algorithm of the SPS for diagnosing of the causative drug in drug-induced cytopenias, using a sample of 40 cases in 39 patients and 85 controls.

## Materials and methods

### Setting and patients

This retrospective, case-control study was conducted at La Paz University Hospital in Madrid, Spain, a tertiary-care teaching facility. Since 2007, all admissions have been monitored by the Proactive Pharmacovigilance Program from Laboratory Signals in Hospital to proactively detect serious ADRs (Ramirez et al., 2010). The study, conducted between 2018 and 2024, investigated thirty-nine suspected cases of drug-induced cytopenia detected by the pharmacovigilance program or through consultations from other specialties within the Pharmacovigilance Unit of the Clinical Pharmacology Department.

The study was approved by La Paz University Hospital Ethics Committee (Code PI-3226; 25 May 2018). Due to the retrospective nature, the study was exempt from informed consent requirements. For all patients initially categorised as having suspected drug-induced cytopenias (neutropenia, agranulocytosis, bone marrow aplasia or haemolytic anaemia), a complete report was submitted to the pharmacovigilance centre in Madrid, Spain (https://www.notificaram.es).

The inclusion criteria for the blood cytopenia cases were: 1) meeting the definition of any type of cytopenia (neutropenia, agranulocytosis, haemolytic anaemia, bicytopenia or pancytopenia), 2) prior drug intake before the blood cytopenia index date, 3) reasonable exclusion of all alternative causes according to the SPS algorithm (Aguirre and García, 2016), and 4) at least one drug had a SPS score ≥+4. Medication errors were excluded from the study.

For agranulocytosis cases, the following criteria standardized by Benichou et al. (Benichou and Solal Celigny, 1991) were also applied: 5) onset of agranulocytosis occurred during treatment or within 7 days of the previous intake of the same drug and no clinical features and more than 1.5 × 10^9^ neutrophils/mm^3^, 1 month after drug discontinuation; 6) the absence of the exclusion criteria “history of congenital or immune-mediated neutropenia,” “recent infection (especially viral),” “prior chemotherapy or radiotherapy” or “therapy with biologicals, haematological disease” and, 7) recurrence of neutropenia or agranulocytosis after repeated treatment with the drug (positive rechallenge).

Age- and sex-matched patients who completed the drug therapy without experiencing any adverse reactions during the study period served as tolerant controls.

Patient safety was ensured by promptly discontinuing the suspected drug(s) after excluding alternative causes, along with close clinical monitoring within the Pharmacovigilance Unit. When drug re-exposure was clinically indicated, it was performed under strict medical supervision with appropriate laboratory follow-up.

### Case detection, definition and severity criteria

The procedure of the pharmacovigilance program for detecting drug-induced cytopenia cases has been described elsewhere (Ramirez et al., 2010). Briefly, in phase I, on-file laboratory data at admission or during hospitalisation were screened 7 days a week, 24 h a day, for results of abnormally low values of blood cell count. In phase II, the patients were identified to avoid duplicates, and electronic medical records were reviewed. In those cases, where low blood cell count was clearly attributable to other alternative causes, the patients were not further analysed because a drug-induced cytopenia was unlikely. In phase III, a case-by-case evaluation was performed for the remaining cases. When the drug history was unclear, we interviewed the patients or their relatives to obtain more details and conducted additional tests.

For each case, the index day was defined as the earliest occurrence of either the onset of clinical symptoms or the detection of cytopenia in blood tests.

Once ruled out alternative causes for blood cytopenias, the suspicious drugs were withdrawn after discussion with the attending physician and patients were offered to be followed in the Pharmacovigilance Unit.

The case definition of the different types of IDIN relied on the following clinical chemistry criteria: 1) neutropenia: ANC < 1.5 × 10^3^/µL, Hb ≥ 10.0 g/dL and APC ≥ 0.1 × 10^6^/µL; 2) agranulocytosis was defined as ANC < 0.5 × 10^3^/µL, Hb ≥ 10.0 g/dL and APC ≥ 0.1 × 10^6^/µL; 3) haemolytic anaemia: Hb < 6.5 g/dL with reticulocyte count > 0.105 × 10^6^/µL; LDH levels > 190 UI/L, UCB > 1.2 mg/dL, WBC count ≥ 3.5 × 10^3^/µL and APC ≥ 0.05 × 10^6^/µL; 4) bicytopenia: WBC count ≤ 3.5 × 10^3^/µL and one of the following: Hb < 10.0 g/dL or APC ≤ 0.05 × 10^6^/µL; 5) pancytopenia: WBC count ≤ 3.5 × 10^3^/µL, Hb ≤ 10.0 g/dL, APC ≤ 0.05 × 10^6^/µL.

### Causality assessment

The causality assessment was performed using the causality algorithm of the SPS (Aguirre and García, 2016) (Supplementary Figure S1). This algorithm evaluates the causal relationship between a suspected drug and ADR. The SPS algorithm consists of 7 questions or criteria: 1) time between the start of treatment and ADR onset; 2) prior evidence in the literature regarding the relationship between the drug and the ADR; 3) ADR evolution after drug withdrawal; 4) effect of re-exposure; 5) presence of non-drug or other drug related cause; 6) presence of contributing factors favouring causality relationship, and 7) results of tests supporting drug causality.

The total score (ranging from −8 to +11) from the domain-specific assessment classifies the drug into 5 separate categories: definite (≥8), probable (6–7), possible (4–5), conditional (1–3) or unrelated (<0). A SPS score ≥+4 was considered drug-related.

Drugs implicated in the causation of drug-induced blood cytopenias were classified into therapeutic subgroups according to the Anatomical Therapeutic Chemical (ATC) system. Latency periods reported in the literature were recorded and compared with observed latencies.

### *In vitro* lymphocyte transformation test

LTT was performed using different concentrations of the drug(s) involved in the blood cytopenias cases (SPS algorithm score ≥+3) and tolerant controls in order to assess its contribution in enhancing causality assessment, although only drugs with a final score ≥+4 were classified as “suspected culprit drugs” in the analysis. LTT was performed after cytopenia recovery and at least 1 month after steroid therapy was stopped, if applicable.

Lymphocyte proliferation was measured as previously described (Pichler and Tilch, 2004; Vílchez-Sánchez et al., 2020a). Briefly, mononuclear cells were separated over a density gradient (Histopaque-1077, Sigma-Aldrich) from fresh peripheral blood and were plated in flat bottom wells of microtitre plates at 2 × 10^5^ cells/well. Cells were incubated for 6 days with various drug concentrations in triplicate. Drugs were assayed at concentrations of 1, 10 and 100 μg/mL, and occasionally, a lower or higher concentration (0.1, 200 or 500 μg) were used, as previously described (Pichler and Tilch, 2004). We used phytohemagglutinin (5 μg/mL) as a positive control. For the final 18 h of the incubation period, proliferation was determined by adding 1 μCi [3H] thymidine.

All laboratory procedures involving human peripheral blood samples were conducted in accordance with biosafety level 2 (BSL-2) conditions. Institutional protocols were strictly followed for sample handling, storage, and disposal to ensure both staff and environmental safety.

Proliferative responses were expressed as the stimulation index (SI), calculated as the ratio between the mean counts per minute in cultures with the drug and those without drug. A receiver-operating characteristic (ROC) curve analysis was performed using LTT results from 85 drug-tolerant participants to determine the optimal SI cut-off value. An LTT result was considered positive if the SI exceeded the threshold at any drug concentration. Patients were classified as having immune-mediated cytopenia if at least one LTT result was positive.

### Statistical analysis

Continuous variables are presented as mean ± standard deviation (SD) or median with interquartile range (IQR), depending on normality assessed by the Kolmogorov-Smirnov test. A comparison was made between the latency described in the literature and that observed in the study. Categorical variables are expressed in absolute terms and percentages. We employed the chi-squared test to compare the categorical variables and employed Student’s t-test for the continuous variables with a normal distribution. In the event the data did not have a normal distribution, we used the nonparametric Mann-Whitney *U*-test or Kruskal-Wallis test, as appropriate. Differences were considered significant when the *p*-value was <0.05. The strength of the association between female sex and the development of cytopenia was quantified using an Odds Ratio (OR) with its 95% confidence interval (CI). A p-value of less than 0.05 was considered statistically significant. Descriptive analysis was performed using R (Integrated Development for R Studio, PBC, Boston, MA; http://www.rstudio.com, accessed on 19 February 2025).

Receiver-operating characteristic (ROC) curve analysis was performed to determinate the optimal cut-off value for the SI in LTT. This analysis aimed to maximize the sum of specificity and sensitivity in differentiating between cases with a clinical diagnosis of drug-induced blood cytopenia and tolerant controls. Sensitivity analysis was conducted using different SPS scores. These statistical analyses were performed using the IBM SPSS Statistics version 21.0 (IBM Corporation, Armonk, NY, United States).

## Results

### Characteristics of drug-induced cytopenias cases

Of the 39 patients (40 cases) with drug-induced blood cytopenia, the most common types were agranulocytosis (n = 29, 72.5%) and neutropenia (n = 6, 15.0%). Less frequent were haemolytic anaemia (n = 2, 5.0%), bicytopenia (n = 2, 5.0%), and pancytopenia (bone marrow aplasia; n = 1, 2.5%). The median [interquartile range, IQR] age was 38 [23.5–59] years, with no significant age differences between cytopenia types. Most patients were female (n = 26, 66.7%). The distribution of cases across age groups was as follows: minors (0–17 years), 22.5% (9/40); adults (18–65 years), 55.0% (22/40); and older adults (>65 years), 22.5% (9/40). Adult patients accounted for the majority of agranulocytosis (58.6%, 17/29) and neutropenia (50%, 3/6) cases.

Patient characteristics are summarized in Table 1. Most cases presented with three or more comorbidities (n = 26, 66.7%) and reported no previous allergies (n = 28, 71.8%). The median [IQR] of comorbidities per patient was 4 [2–6.5]. Most patients with agranulocytosis (16/28, 57.1%), all patients with neutropenia, and all patients with bicytopenia or bone marrow aplasia had three or more comorbidities.

**TABLE 1:** Characteristics of patients with blood cytopenia^a^.

Variable		Results
Sex, n (%)		
• Female		26 (66.7)
• Male		13 (33.3)
Sex distribution by type of cytopenia		
	Male, n (%)	Female, n (%)
Agranulocytosis	9 (31.0)	20 (69.0)
Neutropenia	2 (33.3)	4 (66.7)
Haemolytic anaemia	0 (0.0)	2 (100.0)
Bicytopenia	2 (100.0)	0 (0.0)
Pancytopenia (bone marrow aplasia)	0 (0.0)	1 (100)
Overall mean age (median [IQR])		38 [23.5–59]
• Agranulocytosis (median [IQR])		31 [23–54]
• Neutropenia (median [IQR])		46 [12.25–57.25]
• Haemolytic anaemia (median [IQR])		59 [48.5–69.5]
• Bicytopenia (median [IQR])		74.5 [70.25–78.75]
• Pancytopenia (bone marrow aplasia) (median [IQR])		60 [60–60]
Chronic conditions, n (%)		
• 1		8 (20.5)
• 2		5 (12.8)
• 3 or more		26 (66.7)
Number of comorbidities (median [IQR])		4 [2–6.5]
History of allergic reactions (including ADRs), n (%)		
• No allergies		28 (71.8)
• 1 allergy		7 (17.9)
• 2 or more allergies		4 (10.3)
Number of allergies (median [IQR])		0 [0–1]
Type of cytopenia		
• Agranulocytosis		29 (72.5)
• Neutropenia		6 (15.0)
• Haemolytic anaemia		2 (5.0)
• Bicytopenia		2 (5.0)
• Pancytopenia (bone marrow aplasia)		1 (2.5)
Concomitant reaction, n of patients (%)		
• No		20 (51.3)
• Yes		19 (48.7)
➢ Nonspecific elevation of levels of transaminase and LDH		2 (9.1)
➢ Pneumonia due to COVID-19		2 (9.1)
➢ Pulmonary embolism		2 (9.1)
➢ Acute gingivitis		1 (4.5)
➢ Acute respiratory failure with hypoxia		1 (4.5)
➢ Acute tonsillitis		1 (4.5)
➢ Cholangitis		1 (4.5)
➢ COVID-19		1 (4.5)
➢ Drug rash with eosinophilia and systemic symptoms syndrome		1 (4.5)
➢ Elevation of levels of liver transaminase levels		1 (4.5)
➢ Evans syndrome		1 (4.5)
➢ Herpesviral infection, unspecified		1 (4.5)
➢ Localized skin eruption due to drugs and medicines taken internally		1 (4.5)
➢ Pneumonia, unspecified organism		1 (4.5)
➢ Toxic gastroenteritis and colitis		1 (4.5)
➢ Toxic liver disease with acute hepatitis		1 (4.5)
➢ Toxic liver disease with cholestasis		1 (4.5)
➢ Ulcerative colitis, unspecified, with complications		1 (4.5)
➢ Urinary tract infection, site not specified		1 (4.5)
Symptoms, n (%)		
• No		5 (12.5)
• Yes		35 (87.5)
➢ 2 or more symptoms		29 (72.5)
➢ 1 symptom		6 (15.0)
➢ No symptoms		5 (12.5)
Number of symptoms (median [IQR])		3 [1–4]
Complications, n (%)		
• Non-systemic infection		3 (50.0)
• Anaemisation		2 (33.3)
• Sepsis		1 (16.7)
Outcome, n of patients (%)		
• Recovery		39 (100)
• Recovery with sequelae		0
• Death		0

Abbreviations: IQR, interquartile range; ADRs: adverse drug reactions; COVID-19: Coronavirus Disease 2019; LDH, lactic acid dehydrogenase.

^a^ References: Andersohn et al. (2007), Andres et al. (2009), Kaufman et al. (2006), Maquet et al. (2024), Ramirez et al. (2010).

Most patients (87.5%) presented with symptoms, with the majority (72.5%) experiencing two or more symptoms. The median number of symptoms per case was 3. The most common symptoms were fever (80.0%), nausea and vomiting (25.7%), general discomfort (20.0%), and cutaneous lesions (14.3%). Nearly half of the patients (48.7%) experienced concomitant reactions. Complications included non-systemic infections (3 cases), sepsis (1 case), and anaemia (2 cases).

In our study, 72.5% of patients demonstrated unequivocal agranulocytosis, with a median [IQR] neutrophil count at index date of 0.25 × 10^3^/µL [0.03 × 10^3^/µL - 0.61 × 10^3^/µL] at the nadir of neutrophil decline. Thirty percent had neutrophil counts below 0.1 × 10^9^/L, and 15.0% experienced complications, including local infections or sepsis. Among those with neutropenia, 12.5% did not exhibit clinical signs of infection, similar to patients with other forms of cytopenia. Fever was the most common symptom, occurring in 80.0% of cases, while 12.5% remained asymptomatic.

### Temporal characteristics of drug-induced blood cytopenias

The observed median [IQR] time from drug intake to observation of blood tests alterations (latency) for all cytopenia cases was 8 [3.5–21] days. For those with neutropenia, agranulocytosis, haemolytic anaemia, bicytopenia and the bone marrow aplasia case, the observed median [IQR] latency was 6 [6–12.5], 11 [3–21.25], 4 [4–4], 6.5 [6.25–6.75] and 241 [241–241] days, respectively.

The literature-reported median latency [IQR] (Table 2) for all cytopenia types was 7 [2–15] days. Specifically, the median latency [IQR] was 15 [2–15] days for neutropenia and 7 [2–13.5] days for agranulocytosis. Mean latency data for haemolytic anaemia, bicytopenia, and bone marrow aplasia were unavailable. The median difference [IQR] between observed and literature-reported latency was 0 [-4 to 6] days for all cytopenias, 4 [0–5] days for neutropenia, and 0 [-4 to 6] days for agranulocytosis. No statistically significant differences were found between observed and literature-reported latency.

**TABLE 2:** Median latency to onset and duration of blood cytopenia episodes.

Variable	Results
Overall observed median latency [IQR], days	8 [3.5–21]
Overall described median latency [IQR], days^a^	7 [2–15]
Overall median difference between observed and described latency [IQR], days	0 [-4–6]
Overall median duration of cytopenia [IQR], days	7 [5–16.5]
Overall median latency between onset of cytopenia and date of LTT [IQR], days	180.5 [137.75–279.75]
• Pancytopenia (bone marrow aplasia)	90 [90–90]
• Bicytopenia	63.5 [34.25–92.75]
• Haemolytic anaemia	27.5 [25.75–29.25]
• Neutropenia	7 [6.25–36.25]
• Agranulocytosis	6 [5–12]

Abbreviations: IQR, interquartile range; LTT, lymphocyte transformation test.

^a^ References: Andersohn et al. (2007), Andres et al. (2009), Kaufman et al. (2006), Maquet et al. (2024), Ramirez et al. (2010).

The median [IQR] time to haematological recovery (defined as WBC count ≥ 3.5 × 10^3^/µL; ANC ≥ 1.5 × 10^3^/µL; Hb > 10.0 g/dL; APC ≥ 0.1 × 10^6^/µL) for all cytopenias cases was 7 [5, 16.5] days. Recovery times varied across cytopenia types, ranging from a median of 6 days for agranulocytosis to 90 days for bone marrow aplasia. However, no statistically significant differences in recovery time were observed among the different types of cytopenia (*p* = 0.201).

The median [IQR] time between the onset of cytopenia and the performance of the LTT was 180.5 [137.75–279.75] days for all cases. This time varied across cytopenia types, ranging from 158 [124–272] days for agranulocytosis to 324 [249–399] days for haemolytic anaemia (Table 2). However, no statistically significant differences were observed among the different types of cytopenia (*p* = 0.526).

### Aetiological and diagnostic findings

Microbiology tests were performed on 39 of the 40 patients, with 45.0% (18 cases) yielding a positive result (Table 3).

**TABLE 3:** Microbiological profile of patients with blood cytopenia^a^.

Variable	Results
Microbiology tests, n (%)	
• Negative	21 (52.5)
• Positive	18 (45.0)
• Not performed	1 (2.5)

By type of microbiology test		
Number of positive tests (n [%])	Result	Type of cytopenia
➢ Serology		
1 (25.0)	*Borrelia Burgdorferi*	Agranulocytosis
1 (25.0)	HIV	Agranulocytosis
1 (25.0)	*Mycoplasma* IgG	Haemolytic anaemia
1 (25.0)	EBV IgM	Agranulocytosis
➢ Blood culture, number of positive tests (n [%])		
1 (33.3)	*Escherichia coli*	Bicytopenia
1 (33.3)	*Lactobacillus rahmnosus*	Neutropenia
1 (33.3)	*Streptococcus parasanguinis* and *Neisseria subflava*	Agranulocytosis
➢ Urine culture, number of positive tests (n [%])		
1 (20.0)	*Candida albicans, Enterococcus faecalis, Pseudomonas aeruginosa*	Haemolytic anaemia
3 (60.0)	*Escherichia coli*	Agranulocytosis
1 (20.0)	*Pseudomonas aeruginosa* and *Enterococcus faecium*	Neutropenia
➢ PCR, number of positive tests (n [%])		
1 (25.0)	*Clostridioides difficile* in feces, COVID-19	Agranulocytosis
2 (50.0)	COVID-19	Agranulocytosis
1 (25.0)	HHV-8 and EBV	Agranulocytosis

Abbreviations: COVID-19: Coronavirus Disease 2019; PCR, Polymerase Chain Reaction; HIV, Human Immunodeficiency Virus; HHV-8, Human Herpesvirus 8; EBV, Epstein-Barr Virus; IgG, Immunoglobulin G; IgM, Immunoglobulin M.

^a^ References: Andersohn et al. (2007), Andres et al. (2009), Kaufman et al. (2006), Maquet et al. (2024), Ramirez et al. (2010).

The positive findings revealed a range of viral and bacterial infections across different test types. Serology and PCR tests identified pathogens such as HIV, Epstein-Barr Virus (EBV), and COVID-19. Notably, all four positive PCR results occurred in patients with agranulocytosis. Bacterial infections were also detected, primarily in blood and urine cultures, with *Escherichia coli* being the most frequently isolated pathogen.

ANCA testing was performed in 15/40 (37.5%) cases. Of these, 2 cases (5.0%) were positive, both of which were agranulocytosis cases. The remaining 13 cases (32.5%) were negative. ANCA testing was conducted in cases of agranulocytosis (11 tests, 2 positive), haemolytic anaemia (2 tests, both negative), and neutropenia (2 tests, both negative). No statistically significant differences in ANCA tests results were found among the different types of cytopenia (*p* = 0.591) (Table 4).

**TABLE 4:** ANCA determination and bone marrow examination in patients with blood cytopenia^a^.

Variable	Results
ANCA determination, n (%)	
• Positive	2 (5.0)
• Negative	13 (32.5)
• Not performed	25 (62.5)
Bone marrow examination, n (%)	
• Normal	2 (5.0)
• Anomalous	6 (15.0)
• Not performed	32 (80.0)

Number of positive tests (n)	Result	Type of cytopenia (n)
5	Hypoplasia of granulopoietic cells	Agranulocytosis (4) Bone marrow aplasia (1)
1	Hypoplasia of hematopoietic cells	Bone marrow aplasia (1)
1	Hypoplasia of megakaryocytes	Bone marrow aplasia (1)
1	Hyperplasia of hematopoietic cells	Agranulocytosis (1)
3	Hyperplasia of megakaryocytes	Agranulocytosis (3)

Abbreviations: ANCA, anti-neutrophil cytoplasmic antibodies.

^a^ References: Andersohn et al. (2007); Andres et al. (2009), Kaufman et al. (2006), Maquet et al. (2024), Ramirez et al. (2010).

### Treatment and resolution of drug-induced blood cytopenias

Treatment was administered in 28/40 (70.0%) cases of cytopenia, most frequently for agranulocytosis (20 cases). Half of all cases (20/40, 50.0%) received granulocyte colony-stimulating factor (G-CSF) or granulocyte-macrophage colony-stimulating factor (GM-CSF) therapy, primarily for agranulocytosis (17 cases), followed by neutropenia (2 cases) and bicytopenia (1 case). Corticosteroids were administered in 15/40 (37.5%) cases, mostly in agranulocytosis (9 cases). Immunosuppressants (cyclosporine) were used in only one case (2.5%), which was a bone marrow aplasia case. Four cases (10.0%) received biological therapy, including normal human immunoglobulin (2 cases), mepolizumab (1 case), and rituximab (1 case). Further details on treatment modalities are provided in Table 5.

**TABLE 5:** Therapy in patients with blood cytopenia^a^.

Variable	Results
Therapy for the cytopenia episode, n (%)	
• No	12 (30.0)
• Yes	28 (70.0)

Type of therapy	Number of cases	Type of cytopenia (n)
G-CSF or GM-CSF	17	Agranulocytosis
	2	Neutropenia
	1	Bicytopenia
Corticosteroids	9	Agranulocytosis
	3	Neutropenia
	2	Haemolytic anaemia
	1	Bone marrow aplasia
Biologic therapy	2	Agranulocytosis
	1	Haemolytic anaemia
Immunosuppressive drugs	1	Bone marrow aplasia

Abbreviations: G-CSF, granulocyte colony-stimulating factor; GM-CSF, granulocyte-macrophage colony-stimulating factor.

^a^ References: Andersohn et al. (2007), Andres et al. (2009), Kaufman et al. (2006), Maquet et al. (2024), Ramirez et al. (2010).

The median [IQR] time to resolution for cytopenia cases varied depending on the treatment received, if G-CSF/GM-CSF were administrated was 5 [4.5–7] days, no G/GM-CSF was 18.5 [7–46] days. This difference was statistically significant (p < 0.001), suggesting that treatment with G-CSF or GM-CSF was associated with faster resolution.

Regarding corticosteroids, the median [IQR] time to resolution for cytopenia cases was 7 [5.5–19.50] days when corticosteroids were used and 6 [5–15] days when there were not used. This difference was not statistically significant (*p* = 0.652).

For biological therapies, two cases were treated with intravenous immunoglobulin (IgIV), one of agranulocytosis and one of haemolytic anaemia (concomitant rituximab use), one case of agranulocytosis was treated with mepolizumab. The resolution time for these cytopenias had a median [IQR] of 7 [6–19] days when biological therapies were used, and 7 [5–16] days when they were not, with no statistically significant differences (p = 0.892). Additionally, one case of pancytopenia was treated with cyclosporine, the time of resolution was 90 days.

### Haematological finding

The median [IQR] white blood cell (WBC) count at the index date varied across cytopenia types, ranging from 1.28 × 10^3^/µL [1.09 × 10^3^/µL, 1.48 × 10^3^/µL] in bicytopenia cases to 13.98 × 10^3^/µL [11.79 × 10^3^/µL, 16.16 × 10^3^/µL] in haemolytic anaemia cases. However, there were no statistically significant differences in WBC count among the different types of cytopenia (p = 0.060).

In contrast, the absolute neutrophil count (ANC) at the index date showed significant differences among cytopenia types (p = 0.001). The median [IQR] ANC was lowest in agranulocytosis cases (0.27 × 10^3^/µL [0.04 × 10^3^/µL, 0.41 × 10^3^/µL]) and highest in haemolytic anaemia cases (12.20 × 10^3^/µL [10.13 × 10^3^/µL, 14.26 × 10^3^/µL]). Similarly, the absolute platelet count (APC) at the index date differed significantly among cytopenia types (p = 0.045), with the lowest median [IQR] APC observed in bone marrow aplasia (0.01 × 10^6^/µL [0.01 × 10^6^/µL, 0.01 × 10^6^/µL]). Hemoglobin (Hb) levels at the index date also showed significant differences among cytopenia types (p = 0.024). Haemolytic anaemia cases had the lowest median [IQR] Hb level (5.55 mg/dL).

The median [IQR] minimum WBC count varied across cytopenia types, with no statistically significant differences observed (p = 0.209). However, significant differences were found in the minimum ANC (p = 0.006), APC (p = 0.021), and Hb level (p = 0.038) among the different types of cytopenia. Notably, only 2/29 (6.9%) agranulocytosis cases had an ANC <0.01 × 10^3^/µL.

Eosinophilia was observed in only 2/40 (5.0%) cytopenia cases, both of which were agranulocytosis. There were no statistically significant differences in the presence of eosinophilia (p = 0.939) or the maximum eosinophil count (p = 0.815) among the different types of cytopenia.

Detailed haematological values for each cytopenia case, including WBC counts, are presented in Supplementary Table S2.

### Culprit drugs

The median [IQR] number of drugs studied per patient was 2 [2–3]. In all 40 cytopenia cases, at least one drug had an SPS score +4 or higher, indicating a high level of suspicion for drug causality. Ninety-nine drugs were evaluated, and the majority (n = 90, 90.9%) were suspected to be involved in the blood cytopenia. The median [IQR] SPS score was 6 [5–7]. Of them, 32 (32.3%) had SPS score in the *possible* range (4–5) category, 54 (54.5%) in the *probable* (6–7) category, and 4 (4.0%) in the *definite* (≥8) category. Only 7.1% (n = 7) of drugs had a SPS score below +4, and in two cases, the score could not be determined.

The most frequently evaluated drug by SPS score was metamizole (17.2%), followed by acetaminophen (9.1%) and the combination of amoxicillin and a beta-lactam (8.1%) (Table 6).

**TABLE 6:** Most frequently evaluated drugs.

Drug	Cases, n (%)
Metamizole sodium	17 (17.2)
Acetaminophen	9 (9.1)
Amoxicillin and BLIs	8 (8.1)
Ibuprofen	6 (6.1)
Piperacillin and BLIs	5 (5.1)
Cefixime	4 (4.0)
Amoxicillin	3 (3.0)
Ceftriaxone	3 (3.0)

Abbreviations: BLIs, Beta (ß)-lactamase inhibitor.

The underlying conditions or therapeutic indications for the suspected causative drugs in cases of drug-induced cytopenia are shown in Supplementary Table S3. However, data on the primary pathology of control subjects were not systematically recorded in the source database and were therefore not available for analysis. Nevertheless, all control subjects received the same or similar treatments without developing cytopenias.

In 8 (20.0%) cytopenia cases, only one drug was suspected, while polypharmacy (≥5 drugs) was detected in 2 (5.0%) cases. The distribution of cases by the number of implicated drugs (SPS score ≥+4) is shown in Table 7.

**TABLE 7:** Cytopenia cases by number of drugs ranking ≥+4 by SPS score.

Number of drugs ranking ≥+4 by SPS score	Cases, n (%)
1	8 (20.0)
2	15 (37.5)
3	13 (32.5)
4	2 (5.0)
≥5	2 (5.0)

Abbreviations: SPS, Spanish Pharmacovigilance System.

Most drugs implicated in agranulocytosis (92.1%, n = 70/76), haemolytic anaemia (66.7%, n = 2/3), and all drugs implicated in neutropenia, bicytopenia, and bone marrow aplasia had SPS scores ≥+4. No statistically significant differences in SPS score categories (≥+4 vs. <+4) were found among the different types of cytopenia (p = 0.335).

### LTT as a diagnostic tool for drug-induced cytopenias

**FIGURE 1:**
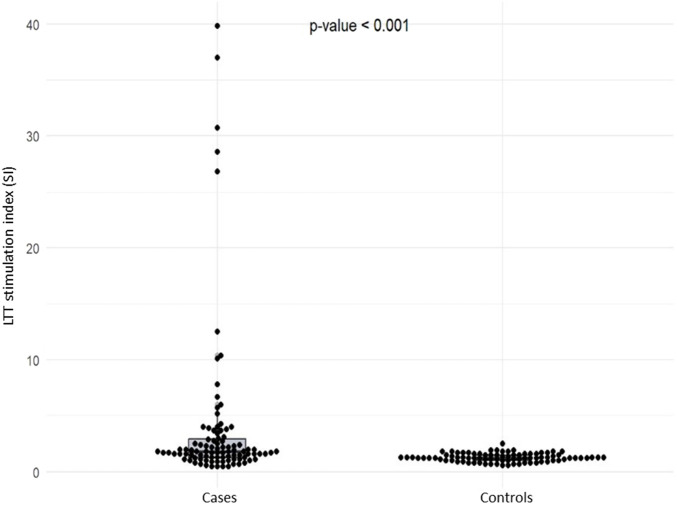
LTT stimulation index in cases and controls.

The controls were paired in age with the cases being the median [IQR] age of cases, 38 [23.5–59], and of controls, 49 [32–61] (p = 0.236). There was no statistically significant difference between the proportion of females between the case group (66.7%) and control group (49.4%), (OR: 2.048; 95% CI: 0.929–4.512). The LTT stimulation index (SI) was significantly higher in cases of drug-induced cytopenias compared to tolerant controls (median [IQR]: (1.80 [1.30–2.90]) vs. 1.20 ([1.00–1.50]; *p* < 0.001) (Figure 1). Conversely, only 1 of 85 (1.2%) controls, who had received rituximab, exhibited a positive LTT (Supplementary Table S1).

**FIGURE 2:**
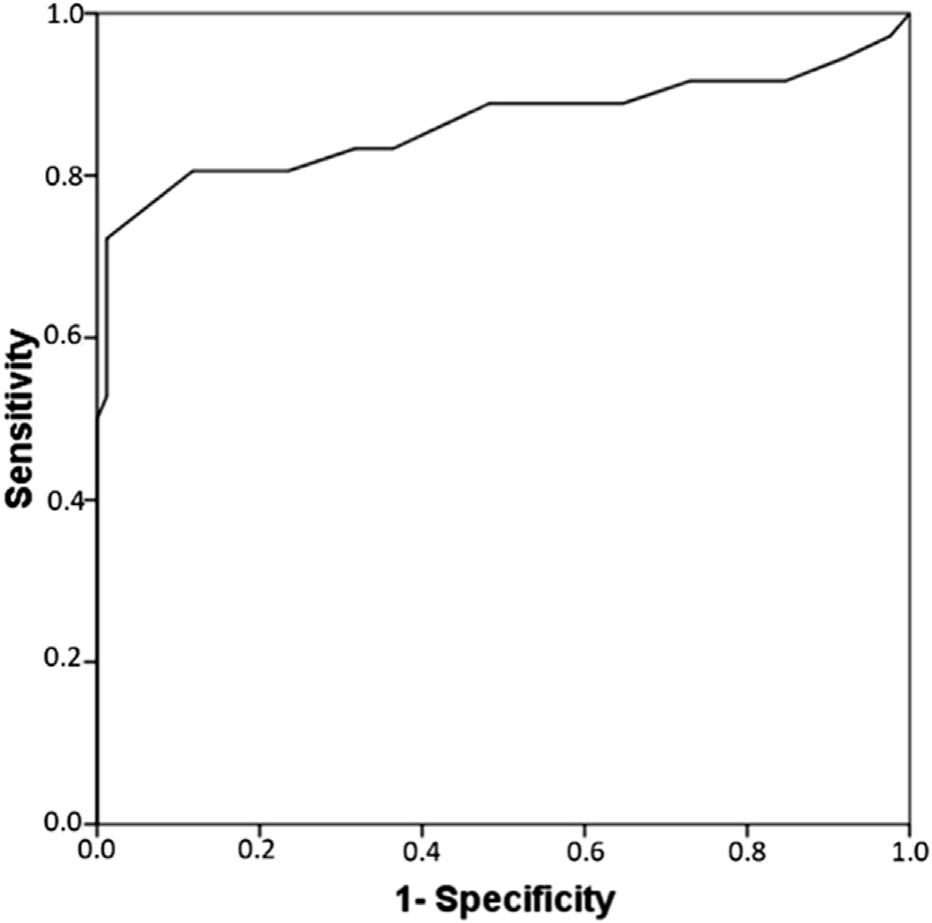
Receiver-operating characteristic curve analysis for the stimulation index generated between patients (*n* = 39) and exposed controls (*n* = 85). An optimal threshold of 1.95 was obtained (area under the curve [AUC], 0.86; 95% asymptotic confidence interval [CI], 0.77–0.96; *p* < 0.001).

To determine the best discriminative threshold for SI, a ROC curve analysis was performed using the 40 cases (in 39 patients) with clinically diagnosed blood cytopenia and 85 tolerant controls. In cases in where multiple drugs were suspected, the maximum SI was used for the analysis. An optimal SI cut-off of 1.95 was identified (Figure 2) yielding a sensitivity of 72% and specificity of 99% (area under the curve [AUC], 0.86; 95% asymptotic confidence interval [CI], 0.77–0.96; *p* < 0.001).

Using this threshold, 41.4% (41/99) of the suspected drugs resulted in a positive LTT, and at least one LTT was positive for 75.0% (30/40) of cases, indicating a drug-specific immune response mechanism underlying the cytopenia. Regarding the number of positive LTTs per case, 60% (24/40) had only one drug inducing a positive proliferative response, 15% (6/40) had two or more drugs (Table 8).

**TABLE 8:** Cytopenia cases by number of positive LTTs.

Number of positive LTTs	Cases, n (%)
0	10 (25.0)
1	24 (60.0)
2	3 (7.5)
3	2 (5.0)
4	0 (0.0)
≥5	1 (2.5)

Abbreviations: SPS, Spanish Pharmacovigilance System.

### Drug subgroup implication and LTT positivity

Analysis of drug involvement, categorized by ATC classification, revealed that in our sample antibacterials (J01) 37.4% (n = 37), analgesics (N02) 26.3% (n = 26) and anti-inflammatory and antirheumatic products (M01) 10.1% (n = 10) were the most frequently implicated drug subgroups in the blood cytopenia cases. A substantial proportion of the implicated drugs (42.4%) showed positive lymphocyte transformation test (LTT) results. Positive LTTs were most frequently associated with Antibacterials (J01, 43.2%), analgesics (N02, 38.4%), immunosuppressants (L04, 25%), anti-inflammatory and antirheumatic products (M01, 20%). These four drug subgroups accounted for 69% of all positive LTTs. Notably, these same four ATC groups also had the highest number of LTTs performed. There were no significant differences in the frequency of positive LTTs among the ATC drug groups (*p* = 0.397). Table 9 provides a detailed breakdown of the number of drugs, SPS scores, and the number of positive LTTs by ATC group.

**TABLE 9:** Number of drugs, SPS score and number of positive LTTs by ATC groups.

Drug subgroups (ATC classification)	Drugs, n (%)	SPS score (median [IQR])	No. of positive LTTs per subgroup/Total no. of positive LTTs (%)
J01 - antibacterials for systemic use	37 (37.4)	6 [5.25–6.75]	16/41 (39.0)
N02 – Analgesics	26 (26.3)	6 [4–7]	10/41 (24.4)
M01 – NSAID (anti-inflammatory and anti-rheumatic products)	10 (10.1)	6 [5–6]	2/41 (4.8)
L04 – Immunosuppressants	4 (4.0)	5.5 [4.75–6]	1/41 (2.4)
A02 – Drugs for acid related disorders	3 (3.0)	5 [4.5–6]	0/41 (0.0)
A07 – Antidiarrheals, intestinal anti-inflammatory/anti-infective agents	3 (3.0)	7 [7–7]	2/41 (4.8)
C03 – Diuretics	3 (3.0)	6.5 [6.25–6.75]	2/41 (4.8)
N05 – Psycholeptics	3 (3.0)	5.5 [5.25–5.75]	2/41 (4.8)
J05 – Antivirals for systemic use	2	6.5 [6.25–6.75]	1/41 (2.4)
L01 – Antineoplastic agents	2	5.00 [5–5]	2/41 (4.8)
P01 – Antiprotozoals	2	6 [6–6]	1/41 (2.4)
B01 – Antithrombotic agents	1	7 [7–7]	1/41 (2.4)
H03 – Thyroid therapy	1	7 [7–7]	1/41 (2.4)
N06 – Psychoanaleptics	1	4 [4–4]	0/41 (0.0)
R05 – Cough and cold preparations	1	6 [6–6]	1/41 (2.4)

Abbreviations: ATC, Anatomical Therapeutic Chemical.

### Number and results of LTTs performed by drug in cases and controls

A detailed summary of all drugs tested by LTT both in cases and controls, including the number of tests performed in each group for each drug, as well as the absolute terms and percentages of positive and negative results, is provided in Supplementary Table S4.

### Relation between SPS score and result of LTT

Among the 41 positive LTTs, 40 (97.6%) had a previous SPS score ≥+4 while only one (2.4%) had a previous SPS score <+4. On the other hand, among negative LTTs, 10.3% (6/58) had a prior SPS score <+4 and 89.7% (52/58) had a score ≥+4. No significant differences in the frequency of positive LTTs depending on the previous SPS score (≥+4 or <+4) were found (*p* = 0.285). Table 10 lists the specific drugs implicated in each case of blood cytopenia, along with their SPS scores and LTT results.

**TABLE 10:** Drugs involved in drug-induced blood cytopenias.

Case	Drugs	SPS score	LTT (SI)	No. of drugs assessed	No. of positive LTTs
1	Ibuprofen Azithromycin Naproxen	6 6 6	**2.50** 1.60 1.10	3	1
1	Metamizole	6	1.70	1	0
2	Mesalazine Amoxicillin/clavulanate Metamizole	7 7 7	**3.70** 1.70 0.50	3	1
3	Ibuprofen Diazepam	4 4	1.30 0.90	2	0
4	Furosemide Amoxicillin/clavulanate	4 4	**5.70** **2.00**	2	2
5	Amoxicillin/clavulanate Ibuprofen Metamizole Acetaminophen	6 3 2 3	**2.20** 1.60 1.00 0.50	4	1
6	Mesalazine Metamizole	4 4	**2.50** 1.40	2	1
7	Piperacillin/tazobactam Metamizole	6 6	**12.50** 1.80	2	1
8	Trimethoprim Sulfamethoxazole Metamizole	6 6 6	**2.20** 1.50 1.50	3	1
9	Metamizole Dexketoprofen Amoxicillin Amoxicillin/clavulanate	6 6 NAVU 4	**2.00** 1.50 1.20 0.80	4	1
10	Fosfomycin Ciprofloxacin Cefuroxime	4 5 5	1.40 1.30 1.10	3	0
11	Metamizole Piperacillin/tazobactam Omeprazole	5 5 4	**3.10** 1.90 1.80	3	1
12	Thiamazole	7	**3.90**	1	1
13	Citalopram Hydroxychloroquine Tocilizumab Acetaminophen Azithromycin	4 6 6 4 6	NAVU 1.70 1.80 1.70 1.50	5	0
14	Hydroxychloroquine Furosemide Codeine Tocilizumab Risperidone	6 6 6 6 6	**7.80** **5.20** **4.00** **3.70** **2.60**	5	5
15	Cefixime Metamizole	5 4	1.30 1.00	2	0
16	Acetaminophen	4	0.80	1	0
17	Amoxicillin/clavulanate Amoxicillin	7 NAVU	1.70 **2.10**	2	1
18	Vancomycin Teicoplanin	6 6	1.30 0.70	2	0
19	Metamizole Acetaminophen Amoxicillin/clavulanate Ibuprofen	3 8 8 2	**4.30** **4.00** **3.40** 1.90	4	3
20	Metronidazole Acetaminophen Metamizole Cefuroxime	6 8 6 7	**26.8** 0.70 0.50 0.50	4	1
21	Sulfamethoxazole/trimethoprim	7	**2.40**	1	1
22	Metamizole Famotidine	10 7	**37.00** NA	2	1
23	Metamizole Azathioprine Piperacillin/tazobactam	4 4 4	**2.40** 0.60 **6.00**	3	2
24	Cefixime Dexketoprofen	7 7	**2.30** 1.00	2	1
25	Lorazepam Nirmatrelvir and ritonavir	5 7	**4.00** **2.80**	2	2
26	Cefixime	6	**10.10**	1	1
27	Naproxen Acetaminophen	5 3	**6.70** 1.60	2	1
28	Piperacillin/tazobactam Ceftriaxone	6 7	0.90 0.70	2	0
29	Piperacillin/tazobactam Metamizole	7 7	**10.40** 1.10	2	1
30	Metamizole Ibuprofen Acetaminophen	7 7 3	**2.00** 1.10 0.90	3	1
31	Linezolid	6	**2.90**	1	1
32	Metamizole Acetaminophen Ceftriaxone	6 5 5	**2.20** NAVU NAVU	3	1
33	Metamizole Amoxicillin/clavulanate Spironolactone	7 7 7	**2.70** 1.80 1.70	3	1
34	Rituximab	5	**3.80**	1	1
35	Edoxaban Rifaximin	7 7	**2.30** 1.60	2	1
36	Valganciclovir Cefixime Micofenolate	6 6 5	1.60 1.80 1.60	3	0
37	Cefotaxime Ceftriaxone Amoxicillin/clavulanate	6 6 6	**28.60** **39.80** **30.70**	3	3
38	Rituximab Acetaminophen Amoxicillin	5 4 4	**3.00** 1.10 1.00	3	1
39	Omeprazole Ibuprofen	5 5	1.80 1.60	2	0

Abbreviations: SPS, Spanish Pharmacovigilance System; LTT, lymphocyte transformation test; NAVU, not available; NA, not assessable. Bold values: positive LTT result (SI).

### Lymphocyte reactivity in relation to blood cytopenia characteristics

Positive LTT results were observed in 79.3% (23/29) of agranulocytosis cases, 66.7% (4/6) of neutropenia cases, and all cases of haemolytic anaemia and bone marrow aplasia, while all cases of bicytopenia were negative.

### Drug tolerance after cytopenia episode

Patients with drugs that elicited a positive LTT result were not intentionally re-challenged with drugs. However, accidental re-exposure occurred in three patients. One of these patients did not tolerate the suspected drug (metamizole) and experienced a recurrence of febrile neutropenia.

On the other hand, for drugs with a final causality score between 4 and 5 (categorized as ‘possible’ in the SPS algorithm) and a negative LTT result, a risk minimization plan with haematological monitoring was recommended if drug re-exposure was considered. Among the 40 cytopenia cases, 23 (57.5%) patients were re-exposed to these drug(s). Notably all re-exposed cases tolerated the drug(s), including 18 (100.0%) cases of agranulocytosis, 3 (100%) cases of neutropenia and 1 (100.0%) case of bicytopenia (Table 11).

**TABLE 11:** Cases with drug re-exposure after a blood cytopenia episode.

Case	Blood cytopenia	Drug with re-exposure	SPS score	LTT result	Tolerance to drug re-exposure
1	Agranulocytosis	Naproxen	6	1.1	Yes
	Agranulocytosis	Metamizole	6	1.7	Yes
2	Agranulocytosis	Metamizole	7	0.5	Yes
3	Agranulocytosis	Ibuprofen	4	1.3	Yes
		Diazepam	4	0.9	Yes
5	Agranulocytosis	Metamizole	2	1.0	Yes
		Acetaminophen	3	0.5	Yes
8	Agranulocytosis	Metamizole	6	1.5	Yes
9	Agranulocytosis	Dexketoprofen	6	1.5	Yes
		Amoxicillin	NAVU	1.2	Yes
		Amoxicillin/Clavulanate	4	0.8	Yes
10	Neutropenia	Fosfomycin	4	1.4	Yes
		Ciprofloxacin	5	1.3	Yes
		Cefuroxime	5	1.1	Yes
13	Agranulocytosis	Citalopram	4	NAVU	Yes
		Acetaminophen	4	1.7	Yes
		Azithromycin	6	1.5	Yes
14	Agranulocytosis	Codeine	6	**4.0**	Yes
16	Agranulocytosis	Acetaminophen	4	0.8	Yes
18	Neutropenia	Vancomycin	6	1.3	Yes
19	Agranulocytosis	Ibuprofen	2	1.9	Yes
20	Agranulocytosis	Acetaminophen	8	0.7	Yes
		Cefuroxime	7	0.5	Yes
22	Neutropenia	Metamizole	10	**37.0**	No (febrile neutropenia)
24	Agranulocytosis	Dexketoprofen	7	1.0	Yes
28	Bicytopenia	Piperacillin/Tazobactam	6	0.9	Yes
		Ceftriaxone	7	0.7	Yes
30	Agranulocytosis	Acetaminophen	2	0.9	Yes
31	Neutropenia	Linezolid	6	**2.9**	Yes
33	Agranulocytosis	Amoxicillin/Clavulanate	7	1.8	Yes (amoxicillin)
36	Agranulocytosis	Micofenolate	5	1.6	Yes
38	Agranulocytosis	Acetaminophen	4	1.1	Yes
39	Agranulocytosis	Omeprazole	5	1.8	Yes
		Ibuprofen	5	1.6	Yes

Abbreviations: SPS, Spanish Pharmacovigilance System; LTT, lymphocyte transformation test; NAVU: not available. Bold values: positive LTT result (SI).

A 100% negative predictive value (NPV), with a 95% confidence interval of 94.4%–100%, was observed for a causality score below 6 combined with a negative lymphocyte transformation test (LTT) result. This indicates that in patients with these findings, drug re-exposure under haematological monitoring is likely safe. This high NPV strongly supports the clinical utility of LTT and causality scoring for accurately predicting drug re-exposure tolerance in haematological cytopenias. It enables clinicians to confidently proceed with re-exposure when appropriate, minimizing the risk of adverse reactions, particularly in severe cases like agranulocytosis or febrile neutropenia, and enhancing overall patient safety.

## Discussion

The International Agranulocytosis and Aplastic Anemia Study (IAAAS), conducted in 1980 with participants from Israel and seven European regions, was a pivotal population-based case-control surveillance study that established the foundation for our current understanding of these conditions (Risks of agranulocytosis and aplastic anemia, 1986). The current diagnostic criteria for blood cytopenias were established by Benichou and colleagues during an international consensus meeting in collaboration with the IAAAS (Benichou and Solal Celigny, 1991; Kaufman et al., 1993).

Aggregated data from the IAAAS, alongside case reports and clinical trials, indicate that the incidence of drug-induced agranulocytosis ranges between 1.6 and 15.4 cases per million individuals annually (Andersohn et al., 2007; Andrès et al., 2017; Salama et al., 1989). The occurrence of drug-related neutropenia increases with age and appears slightly higher in women in population-based and clinical studies (Andrès et al., 2017; Ibáñez et al., 2005; van der Klauw et al., 1999). In our study, we also observed a higher frequency in women (69%) although the rate in older adults was lower than expected, at 17.2%. This lower frequency in elderly patients might be attributed to hospitalization bias and the extended diagnostic process, up to 6 months.

This study emphasizes the involvement of classical causative agents, with antibiotics accounting for 37.4% of cases, particularly β-lactams, followed by analgesics at 26.3%, with metamizole (dipyrone) being the most frequent, and NSAIDs comprising 10.1%. These findings are consistent with previous research on agranulocytosis (Andersohn et al., 2007; Andrès et al., 2011; Curtis, 2014). However, in contrast to earlier reports, our study did not identify any cases related to antithyroid drugs. This discrepancy may be attributed to the exclusion of patients taking antithyroid medications who declined to participate in the causality study. These patients may have considered the causality assessment unnecessary as their need for antithyroid drugs would cease following radioiodine therapy or thyroidectomy.

In our study, metamizole emerged as the leading causative drug. Notably, metamizole is not distributed in Anglo-American countries, including the United Kingdom, Canada, and the United States, nor in Scandinavian countries such as Finland, Denmark, and Sweden. However, it is widely available without restriction in countries like Spain, Russia, Brazil, Mexico, Israel and Germany. (Tomidis Chatzimanouil et al., 2023). The primary cause for this debate is its proclivity to generate agranulocytosis. According to recent evidence, the incidence of metamizole-related agranulocytosis is steady, and the absolute risk of agranulocytosis linked with metamizol at standard doses and for short periods of time is extremely low (Ibáñez et al., 2005; Tomidis Chatzimanouil et al., 2023). The focus of metamizol usage restrictions is now patient education on agranulocytosis symptoms for early detection and immediate treatment cessation when they appear, removing routine blood count recommendations but keeping contraindications for haematological risk patients (AEMPS, 2024).

In this potentially life-threatening condition, contemporary treatment approaches, particularly the use of hematopoietic growth factors such as G-CSF, are likely to enhance patient outcomes. Our cases experienced complete recovery from cytopenia, with no observed mortality or long-term sequelae. Statistically significant reductions in the duration of neutropenia were associated with G-CSF or granulocyte-macrophage colony-stimulating factor (GM-CSF) use, regardless of initial infection status. Similar reductions in neutropenia duration have been demonstrated in other retrospective studies evaluating the effects of G-CSF or GM-CSF treatment (Andrès et al., 2002; Beauchesne and Shalansky, 1999).

Agranulocytosis caused by nonchemotherapy drugs might impact mature neutrophil granulocytes and their bone marrow progenitors (Heit et al., 1985; Salama et al., 1989). In our cases, 62.5% of bone marrow examinations evidence of diminished neutrophil granulocyte production. This percentage is consistent with prior research on drug-induced agranulocytosis caused by drugs other than chemotherapy, which has reported impaired granulopoiesis in 65%–68% of patients (Andersohn et al., 2007; Heit et al., 1985; Ibáñez et al., 2005). The observation of reduced granulocyte precursor cell production in the bone marrow highlights a key pathophysiology distinction between acute agranulocytosis and other drug-induced haematological cytopenias, such as immune thrombocytopenia or haemolytic anaemia, where immune-mediated destruction primarily targets peripheral blood cells (Aster, 2005).

Although the precise pathophysiology of drug-induced blood cytopenias remains incompletely understood, both immunologic and toxic mechanisms have been implicated (Salama et al., 1989; Tomidis Chatzimanouil et al., 2023; Wall et al., 1984). In many situations, neutropenia arises from chronic medication exposure, which leads to diminished granulocyte production due to bone marrow hypoplasia. In other circumstances, repetitive, intermittent exposure, as observed with β-lactams, suggest an immune-mediated process. There is substantial evidence indicating that the majority of these reactions are driven by immune mechanisms (Andrès et al., 2011; Johnston and Uetrecht, 2015; Nyfeler and Pichler, 1997; Rattay and Benndorf, 2021). Historically, the prevailing theory of immune-mediated drug-induced agranulocytosis postulated that the drug, either directly or through a reactive metabolite, forms an irreversible covalent (hapten-like) bond with neutrophil membrane proteins, triggering the development of antibodies or T cells directed against the modified membrane structure. Alternatively, true anti-neutrophil autoantibodies may develop independently of drug presence (Christie, 1993; Curtis, 2014). However, more recent studies have shown that the hapten mechanism does not account for all cases. Drug-specific T lymphocytes can directly recognize drug-modified peptides presented by Major Histocompatibility Complex (MHC) molecules and induce apoptosis of neutrophils or their bone marrow progenitors, leading to cytopenia (Posadas and Pichler, 2007; Uetrecht, 2007). In our study, three patients with agranulocytosis tested positive for anti-neutrophil cytoplasmic antibodies (ANCA). These cases of agranulocytosis were associated with mesalazine, hydroxychloroquine and lorazepam use. ANCA-positive vasculitis has been reported in association with various drugs, including hydralazine, propylthiouracil, and minocycline (Akamizu et al., 2002; Choi et al., 2000; Elkayam et al., 1998; Rueda et al., 2020). The detection of ANCA following drug exposure suggests an immunological response targeting either native or altered myeloperoxidase on neutrophils. However, the direct role of these antibodies in mediating agranulocytosis remains uncertain (Fibbe et al., 1986; Gilligan et al., 1996). Many cytopenias are also associated with eosinophilia, which is also characteristic of a drug hypersensitivity reaction. When eosinophilia is present it is highly suggestive of an immune mechanism (Johnston and Uetrecht, 2015; Ramírez et al., 2017). Nevertheless, in our study only 5.0% of patients presented eosinophilia.

The onset of drug-induced agranulocytosis and neutropenia usually is 1–6 months, though a rapid onset of days to weeks is observed in cases of immunological mechanism involving prior sensitization (Andersohn et al., 2007; Kaufman et al., 2006; Ramirez et al., 2010). Similarly, aplastic anaemia may manifest up to 6 months post-drug exposure, with a minimum latency of 10 days (Kaufman et al., 2006). Drug-induced thrombocytopenia exhibits a shorter latency, with initial exposure leading to symptoms in 5–7 days, and prior sensitization shortening this to 2–3 days (Andres et al., 2009). Haemolytic anaemia presents with a variable latency, ranging from hours to weeks (Maquet et al., 2024). In our study, we observed the following median [IQR] latencies: agranulocytosis, 11.00 [3.00, 21.25] days; neutropenia, 6.00 [6.00, 12.50] days, bicytopenia 6.50 [6.25, 6.75] days; haemolytic anaemia was 4.00 [4.00, 4.00] days; and bone marrow aplasia, 241.00 [241.00, 241.00] days. These findings align with previously reported data.

SPS algorithm can standardise and support the clinical assessment of drug-induced cytopenias. However, a significant limitation arises when multiple suspected drugs yield identical scores, hindering precise causal attribution (Aguirre and García, 2016). Therefore, a tool to assist the SPS-based causality assessment is desirable. Many cytopenias are associated with a positive lymphocyte transformation test because the majority of them are immune mediated (Nyfeler and Pichler, 1997). However, this test is ineffective for identifying the causative drug if the mechanism of cytopenia is a direct toxicity (Ono et al., 1991). Therefore, a multifaceted approach, combining the SPS algorithm with complementary diagnostic tools, is crucial for accurate drug causality determination in cytopenias.

The limitations of the LTT include its dependence on the clinical entity and drug causing the adverse reaction, as well as its lack of standardization, which leads to a high degree of accuracy variability across published studies (Mayorga et al., 2017; 2016). It has been proposed that LTT performs better as a diagnostic tool for mild delayed drug hypersensitivity responses than for severe reactions, such as organ-specific reactions (Mayorga et al., 2017). Quantitatively, the LTT presents an overall mean sensitivity of 56% and a specificity of 94% (Mayorga et al., 2017). This indicates that a positive LTT result is highly reliable for confirming drug hypersensitivity, while a negative result does not reliably exclude it. Noticeably, the positivity threshold for SI obtained by the ROC curve analysis is equivalent to that arbitrarily considered in other studies (SI ≥ 2) (Vílchez-Sánchez et al., 2020a). Based on these findings, we posit that the LTT can be a valuable tool for strengthening causality assessment in cases of suspected drug-induced cytopenias, particularly when a positive result is obtained.

A key strength of this study lies in its prospective pharmacovigilance methodology, designed to effectively detect cases of drug-induced cytopenias. One limitation is the absence of controls for all drugs taken by the cytopenia cases, presenting a potential confounding factor. Nonetheless, there was an inclusion of exposed controls strategically implemented to assess the performance of the LTT within a representative control population. This approach facilitated the determination of a generalized positive threshold, which is often arbitrarily assigned or calculated using unexposed controls. Another limitation of this study is the relatively small sample size, necessitating further validation in a larger, prospective cohort. Despite these limitations, in conclusion our results demonstrate that LTT enhance causality assessments in suspected cases of drug-induced cytopenia cases.

## Data Availability

The raw data supporting the conclusions of this article will be made available by the authors, without undue reservation.
