# Supplementary material for: Impact of lymphocyte transformation test on the diagnostic accuracy of the culprit drug in drug-induced cytopenias: a case-control study
Source: Front Pharmacol. 2025 Dec 5;16:1601450. doi: 10.3389/fphar.2025.1601450 (PMC12715524; doi:10.3389/fphar.2025.1601450)
Supplement: Supplementary file 1 [file Supplementaryfile1.docx]

**Title: Impact of lymphocyte transformation test on the diagnostic accuracy of the culprit drug in drug-induced cytopenias: a case-control study**

**SUPPLEMENTARY MATERIAL**

**FIGURE S1.** *Spanish Pharmacovigilance System (SPS) algorithm.*

*Abbreviations: AR, adverse reaction; ADR, adverse drug reaction; SmPC: Summary of Product Characteristics. *References:* Aguirre et al., 2016.

**TABLE S1.** *Lymphocyte transformation test results in tolerant controls.*

| **Control** | **Age (years)** | **Sex** | **Drug** | **LTT (SI)** |
| --- | --- | --- | --- | --- |
| 1 | 1 | M | Acetaminophen | 1.7 |
| 2 | 3 | M | Acetaminophen | 1.7 |
| 3 | 49 | F | Acetaminophen | 1.5 |
| 4 | 13 | M | Amoxicillin | 1.9 |
| 5 | 2 | F | Amoxicillin | 0.9 |
| 6 | 48 | M | Amoxicillin | 1 |
| 7 | 61 | M | Cefixime | 1.2 |
| 8 | 72 | M | Cefixime | 1.8 |
| 9 | 48 | M | Cefixime | 1.3 |
| 10 | 69 | F | Cefixime | 1.5 |
| 11 | 33 | F | Cefixime | 1.1 |
| 12 | 9 | M | Ceftriaxone | 1.8 |
| 13 | 54 | F | Ceftriaxone | 1.6 |
| 14 | 57 | F | Ceftriaxone | 1.9 |
| 15 | 50 | M | Ceftriaxone | 1.3 |
| 16 | 49 | F | Ceftriaxone | 1.1 |
| 17 | 65 | F | Ceftriaxone | 1.3 |
| 18 | 24 | F | Cefuroxime | 1.6 |
| 19 | 40 | M | Cefuroxime | 1.4 |
| 20 | 26 | F | Cefuroxime | 1.3 |
| 21 | 53 | M | Cefuroxime | 1.5 |
| 22 | 41 | M | Cefuroxime | 1.1 |
| 23 | 20 | F | Cefuroxime | 1.6 |
| 24 | 48 | M | Escitalopram | 0.7 |
| 25 | 60 | M | Escitalopram | 0.9 |
| 26 | 49 | M | Escitalopram | 1.2 |
| 27 | 72 | F | Escitalopram | 0.7 |
| 28 | 47 | F | Hydroxychloroquine | 1.7 |
| 29 | 77 | F | Hydroxychloroquine | 1.2 |
| 30 | 50 | M | Hydroxychloroquine | 1.7 |
| 31 | 27 | F | Hydroxychloroquine | 1.6 |
| 32 | 53 | F | Hydroxychloroquine | 1 |
| 33 | 25 | F | Linezolid | 1.4 |
| 34 | 11 | M | Linezolid | 0.8 |
| 35 | 82 | M | Linezolid | 1.2 |
| 36 | 39 | M | Linezolid | 1.1 |
| 37 | 61 | F | Linezolid | 0.9 |
| 38 | 33 | M | Lorazepam | 0.7 |
| 39 | 51 | M | Lorazepam | 1.8 |
| 40 | 57 | M | Lorazepam | 1.3 |
| 41 | 38 | F | Lorazepam | 1 |
| 42 | 40 | M | Lorazepam | 1.3 |
| 43 | 89 | F | Mesalazine | 1.2 |
| 44 | 50 | F | Mesalazine | 0.6 |
| 45 | 79 | M | Mesalazine | 1 |
| 46 | 61 | F | Metamizole | 1.3 |
| 47 | 68 | M | Metamizole | 1.4 |
| 48 | 49 | M | Metamizole | 0.8 |
| 49 | 51 | M | Methimazole | 0.6 |
| 50 | 45 | F | Methimazole | 1.1 |
| 51 | 58 | M | Metronidazole | 1.2 |
| 52 | 51 | M | Metronidazole | 1.6 |
| 53 | 33 | F | Metronidazole | 1.8 |
| 54 | 68 | M | Metronidazole | 1.2 |
| 55 | 32 | M | Metronidazole | 1.2 |
| 56 | 62 | F | Naproxen | 0.8 |
| 57 | 41 | M | Naproxen | 1.2 |
| 58 | 40 | F | Naproxen | 1.5 |
| 59 | 64 | F | Naproxen | 1 |
| 60 | 16 | M | Naproxen | 1.2 |
| 61 | 18 | F | Omeprazole | 1.2 |
| 62 | 4 | M | Omeprazole | 1.1 |
| 63 | 42 | F | Omeprazole | 0.8 |
| 64 | 59 | F | Omeprazole | 1.1 |
| 65 | 66 | F | Omeprazole | 0.7 |
| 66 | 10 | M | Omeprazole | 1.3 |
| 67 | 21 | F | Omeprazole | 1.8 |
| 68 | 39 | M | Piperacillin/Tazobactam | 1.2 |
| 69 | 42 | F | Piperacillin/Tazobactam | 0.9 |
| 70 | 3 | M | Piperacillin/Tazobactam | 1.8 |
| 71 | 57 | M | Piperacillin/Tazobactam | 1.2 |
| 72 | 50 | F | Piperacillin/Tazobactam | 1.5 |
| 73 | 46 | F | Rituximab | 1.9 |
| 74 | 33 | F | Rituximab | **2.5** |
| 75 | 68 | M | Rituximab | 1.5 |
| 76 | 79 | M | Sulfamethoxazole | 1 |
| 77 | 74 | M | Sulfamethoxazole | 0.8 |
| 78 | 20 | F | Sulfamethoxazole | 1.3 |
| 79 | 72 | F | Sulfamethoxazole | 1.7 |
| 80 | 20 | M | Sulfamethoxazole | 0.7 |
| 81 | 69 | F | Vancomycin | 1.4 |
| 82 | 22 | F | Vancomycin | 1.5 |
| 83 | 20 | F | Vancomycin | 1.3 |
| 84 | 55 | M | Vancomycin | 1.2 |
| 85 | 64 | F | Vancomycin | 0.8 |
| *Abbreviations: F, female; M, male; LTT, lymphocyte transformation test; SI, stimulation index.* | | | | |

**TABLE S2.** Haematological values for each cytopenia case.

| **Patient** | **Age, years** | **Sex** | **Blood cytopenia** | **Min ANC (x10^3^/µL)** | **Index ANC (x10^3^/µL)** | **Min ALC (x10^3^/µL)** | **Index ALC (x10^3^/µL)** | **Min Hb (g/dL)** | **Index Hb (g/dL)** |
| --- | --- | --- | --- | --- | --- | --- | --- | --- | --- |
| 1 | 26 | F | A  A | 0.03  0.01 | 0.03  0.03 | 1.42  0.95 | 1.42  0.95 | 10.5  11.4 | 12.7  12.4 |
| 2 | 26 | F | A | 0.04 | 0.04 | 0.6 | 0.7 | 11.5 | 11.5 |
| 3 | 39 | F | A | 0.00 | 0.03 | 2.54 | 2.54 | 12.8 | 13.0 |
| 4 | 83 | M | A | 0.02 | 0.23 | 1.4 | 1.4 | 7.7 | 10.6 |
| 5 | 39 | M | A | 0.19 | 0.19 | 2.0 | 2.7 | 12.9 | 14.8 |
| 6 | 29 | F | A | 0.00 | 0.17 | 0.6 | 0.6 | 8.8 | 9.7 |
| 7 | 41 | F | A | 0.07 | 0.07 | 2.48 | 2.48 | 9.9 | 9.9 |
| 8 | 36 | M | A | 0.00 | 0.37 | 0.7 | 1.9 | 9.3 | 9.3 |
| 9 | 29 | F | A | 0.00 | 0.02 | 0.4 | 0.51 | 8.1 | 10.5 |
| 10 | 55 | M | N | 0.43 | 0.75 | 1.25 | 1.56 | 12.8 | 15.3 |
| 11 | 74 | M | A | 0.08 | 0.08 | 0.77 | 1.09 | 11.3 | 13.0 |
| 12 | 58 | F | A | 0.00 | 0.07 | 2.17 | 2.17 | 11.6 | 14.0 |
| 13 | 57 | F | A | 0.39 | 0.39 | 1.49 | 1.60 | 11.4 | 11.4 |
| 14 | 67 | M | A | 0.41 | 0.41 | 1.98 | 1.98 | 14.0 | 14.0 |
| 15 | 83 | M | B | 0.01 | 0.05 | 0.53 | 0.89 | 9.3 | 11.1 |
| 16 | 0,4 | M | A | 0.30 | 0.47 | 3.79 | 5.82 | 10.0 | 10.0 |
| 17 | 1,5 | F | A | 0.49 | 0.49 | 6.14 | 6.14 | 12.6 | 12.6 |
| 18 | 2 | F | N | 0.88 | 0.93 | 5.56 | 5.56 | 10.5 | 10.5 |
| 19 | 31 | F | A | 0.01 | 0.01 | 0.34 | 0.39 | 10.4 | 14.2 |
| 20 | 3 | F | A | 0.49 | 0.49 | 2.22 | 2.22 | 11.0 | 11.0 |
| 21 | 69 | F | A | 0.36 | 0.41 | 0.91 | 1.10 | 11.0 | 12.4 |
| 22 | 37 | F | N | 0.84 | 0.84 | 2.91 | 3.27 | 11.0 | 11.0 |
| 23 | 58 | F | N | 0.27 | 1.23 | 0.93 | 1.73 | 9.7 | 9.7 |
| 24 | 24 | F | A | 0.17 | 0.37 | 0.90 | 1.77 | 12.9 | 14.0 |
| 25 | 76 | F | A | 0.37 | 0.61 | 1.86 | 2.10 | 10.3 | 11.3 |
| 26 | 80 | F | HA | 2.93 | 16.33 | 6.45 | 18.35 | 3.2 | 6.3 |
| 27 | 38 | F | HA | 3.12 | 8.06 | 3.4 | 9.6 | 4.8 | 4.8 |
| 28 | 66 | M | B | 1.04 | 1.04 | 1.68 | 1.68 | 9.7 | 9.8 |
| 29 | 36 | M | A | 0.22 | 0.53 | 1.01 | 1.01 | 11.5 | 12.5 |
| 30 | 23 | F | A | 0.35 | 0.90 | 1.48 | 2.61 | 13.9 | 14.3 |
| 31 | 67 | M | N | 1.46 | 1.46 | 2.99 | 2.99 | 11.3 | 11.3 |
| 32 | 1 | M | A | 0.16 | 0.27 | 3.32 | 3.32 | 10.5 | 10.5 |
| 33 | 2 | M | A | 0.39 | 0.39 | 4.63 | 4.63 | 12.0 | 12.0 |
| 34 | 54 | F | A | 0.00 | 0.02 | 2.06 | 2.06 | 13.2 | 13.8 |
| 35 | 60 | F | P | 0.55 | 1.82 | 1.58 | 3.37 | 7.3 | 9 |
| 36 | 3 | F | A | 0.36 | 0.36 | 6.49 | 9.1 | 9.0 | 10.7 |
| 37 | 4 | M | N | 0.19 | 1.13 | 2.65 | 2.65 | 9.2 | 9.2 |
| 38 | 45 | F | A | 0.00 | 0.00 | 3.57 | 4.48 | 12.3 | 14.7 |
| 39 | 4 | F | A | 0.16 | 0.35 | 3.20 | 3.52 | 9.2 | 11.2 |
| *Abbreviations: F, female; M, male; ALC: absolute leukocyte count; ANC: absolute neutrophil count; Hb: haemoglobin; APC: absolute platelet count; Min: minimum; Max: maximum; A: agranulocytosis; N: neutropenia; B: bicytopenia; HA: haemolytic anaemia; P: pancytopenia.* | | | | | | | | | |

**TABLE S3.** *Underlying conditions for the drugs associated with cytopenia in patients.*

| **Patient** | **Suspected causative drugs** | **Condition for which the drug was administered** |
| --- | --- | --- |
| 1 | Ibuprofen  Naproxen  Azitromycin | Acute pharyngitis |
|  | Metamizole | Headache |
| 2 | Mesalazine  A/C  Metamizole | Crohn’s disease  Tooth extraction |
| 3 | Ibuprofen  Diazepam | Muscle strain |
| 4 | Furosemide  A/C | Oedema  Liver abscess |
| 5 | A/C | Otalgia |
| 6 | Mesalazine  Metamizole | Ulcerative colitis  Pain |
| 7 | P/T  Metamizole | Episiotomy infection  Myalgia |
| 8 | S/T  Metamizole | Prophylaxis of *Pneumocystis jiroveci* pneumonia  Fever |
| 9 | Metamizole  Dexketoprofen  Amoxicillin  A/C | Tonsillitis |
| 10 | Fosfomycin  Ciprofloxacin  Cefuroxime | Acute pseudomonas pyelonephritis in kidney transplant |
| 11 | Metamizole  P/T  Omeprazole | Necrotising pneumonia  Gastric protector |
| 12 | Thiamazole | Hyperthyroidism caused by amiodarone |
| 13 | HCQ  Azithromycin  Tocilizumab  Acetaminophen  Citalopram | COVID-19  NAVU |
| 14 | HCQ  Furosemide  Codeine  Tocilizumab  Risperidone | COVID-19 pneumonia with secondary PTE  Insomnia |
| 15 | Cefixime  Metamizole | Acute Urinary Retention secondary to Benign Prostatic Hyperplasia |
| 16 | Acetaminophen | UTI |
| 17 | A/C | Acute otitis media |
| 18 | Vancomycin  Teicoplanin | Bacteremia |
| 19 | Acetaminophen  A/C | Acute bacterial pharyngitis |
| 20 | Metronidazole  Acetaminophen  Metamizole  Cefuroxime | Pneumonia |
| 21 | S/T | MRSA isolated in bronchoalveolar lavage |
| 22 | Metamizole  Famotidine | Complex endodontics and COVID-19  CAG |
| 23 | Metamizole  P/T  Azathioprine | Fever following paracentesis  Kidney transplant |
| 24 | Cefixime  Dexketoprofen | Paramandibular infection |
| 25 | Lorazepam  Nirmatrelvir/Ritonavir | Insomnia  COVID-19 |
| 26 | Cefixime | UTI |
| 27 | Naproxen  Acetaminophen | Migraine |
| 28 | P/T  Ceftriaxone | Acute cholangitis with bacteraemia |
| 29 | P/T  Metamizole | Surgical wound infection |
| 30 | Metamizole  Ibuprofen  Acetaminophen | Surgical wound infection |
| 31 | Linezolid | *Mycobacterium abcessus* lung disease |
| 32 | Metamizole  Acetaminophen  Ceftriaxone | COVID-19 |
| 33 | Metamizole  A/C  Spironolactone | Respiratory infection  Proximal tubulopathy |
| 34 | Rituximab | Gastric MALT lymphoma |
| 35 | Edoxaban  Rifaximin | Portal vein thrombosis  Acute diverticulitis |
| 36 | Valganciclovir  Cefixime  Micofenolate | HHV-6 infection  UTI  Kidney transplant |
| 37 | Cefotaxime Ceftriaxone  A/C | Acute otomastoiditis |
| 38 | Rituximab  Acetaminophen  Amoxicillin | Autoimmune sensory axonal polyneuropathy  Respiratory infection |
| 39 | Omeprazole  Ibuprofen | Gastric protector  Surgical wound infection |
| *Abbreviations: LTT, lymphocyte transformation test; A/C, Amoxicillin/Clavulanate; P/T, Piperacillin/Tazobactam; S/T, Sulfamethoxazole/Trimethoprim; HCQ, Hydroxychloroquine; MRSA, methicillin-resistant Staphylococcus aureus; COVID-19, Coronavirus Disease 2019; HHV-6, Human Herpesvirus 6; PTE, pulmonary thromboembolism; CAG, chronic atrophic gastritis; MALT, mucosa-associated lymphoid tissue; UTI, urinary tract infection; NAVU, not available.* | | |

**TABLE S4.** *Number of times drugs were tested by LTT and qualitative results in cases and controls.*

| **Drug** | **Cases** | | | | **Controls** | | | |
| --- | --- | --- | --- | --- | --- | --- | --- | --- |
|  | **Times tested by LTT, n** | **Positive results, n (%)** | **Negative results, n (%)** | **NAVU results, n (%)** | **Times tested by LTT, n** | **Positive results, n (%)** | **Negative results, n (%)** | **NAVU results, n (%)** |
| Acetaminophen | 9 | 1 (11.1) | 7 (77.8) | 1 (11.1) | 3 | 0 (0.0) | 3 (100) | 0 (0.0) |
| Amoxicillin ± Clavulanate | 11 | 5 (45.5) | 6 (54.5) | 0 (0.0) | 3 | 0 (0.0) | 3 (100) | 0 (0.0) |
| Cefixime | 4 | 2 (50.0) | 2 (50.0) | 0 (0.0) | 5 | 0 (0.0) | 5 (100) | 0 (0.0) |
| Ceftriaxone | 3 | 1 (33.3) | 1 (33.3) | 1 (33.3) | 6 | 0 (0.0) | 6 (100) | 0 (0.0) |
| Cefuroxime | 2 | 0 (0.0) | 2 (100) | 0 (0.0) | 6 | 0 (0.0) | 6 (100) | 0 (0.0) |
| (Es)citalopram | 1 | 0 (0.0) | 0 (0.0) | 1 (100) | 4 | 0 (0.0) | 4 (100) | 0 (0.0) |
| HCQ | 2 | 1 (50.0) | 1 (50.0) | 0 (0.0) | 5 | 0 (0.0) | 5 (100) | 0 (0.0) |
| Linezolid | 1 | 1 (100) | 0 (0.0) | 0 (0.0) | 5 | 0 (0.0) | 5 (100) | 0 (0.0) |
| Lorazepam | 1 | 1 (100) | 0 (0.0) | 0 (0.0) | 5 | 0 (0.0) | 5 (100) | 0 (0.0) |
| Mesalazine | 2 | 2 (100) | 0 (0.0) | 0 (0.0) | 3 | 0 (0.0) | 3 (100) | 0 (0.0) |
| Metamizole | 17 | 8 (47.1) | 9 (52.9) | 0 (0.0) | 3 | 0 (0.0) | 3 (100) | 0 (0.0) |
| Methimazole (Thiamazole) | 1 | 1 (100) | 0 (0.0) | 0 (0.0) | 2 | 0 (0.0) | 2 (100) | 0 (0.0) |
| Metronidazole | 1 | 1 (100) | 0 (0.0) | 0 (0.0) | 5 | 0 (0.0) | 5 (100) | 0 (0.0) |
| Naproxen | 2 | 1 (50.0) | 1 (50.0) | 0 (0.0) | 5 | 0 (0.0) | 5 (100) | 0 (0.0) |
| Omeprazole | 2 | 0 (0.0) | 2 (100) | 0 (0.0) | 7 | 0 (0.0) | 7 (100) | 0 (0.0) |
| Piperacillin/  Tazobactam | 5 | 3 (60.0) | 2 (40.0) | 0 (0.0) | 5 | 0 (0.0) | 5 (100) | 0 (0.0) |
| Rituximab | 2 | 2 (100) | 0 (0.0) | 0 (0.0) | 3 | 1 (33.3) | 2 (66.6) | 0 (0.0) |
| Sulfamethoxazole ± Trimethoprim | 2 | 1 (50.0) | 1 (50.0) | 0 (0.0) | 5 | 0 (0.0) | 5 (100) | 0 (0.0) |
| Vancomycin | 1 | 0 (0.0) | 1 (100) | 0 (0.0) | 5 | 0 (0.0) | 5 (100) | 0 (0.0) |

*Abbreviations: LTT, lymphocyte transformation test; HCQ, Hydroxychloroquine; NAVU, result not available.*
